# Extracapillary Proliferation Superimposed on Primary Membranous Glomerulonephritis: A Case Report

**DOI:** 10.7759/cureus.103903

**Published:** 2026-02-19

**Authors:** Oumaima El Kaoua, Nabil Hamouche, Mariam Chettati, Wafaa Fadili, Inass Laouad

**Affiliations:** 1 Department of Nephrology, Centre Hospitalier Universitaire (CHU) Mohammed VI Marrakech, Marrakesh, MAR; 2 Department of Nephrology, Mohammed VI University Hospital, Marrakesh, MAR; 3 Department of Nephrology, Mohammed VI University Hospital of Marrakesh, Faculty of Medicine and Pharmacy of Marrakesh, Cadi Ayyad University, Marrakesh, MAR

**Keywords:** chronic hemodialysis, crescent, immunosuppression, membranous nephropathy, nephrotic syndrome, primary membranous nephropathy, renal failure

## Abstract

We report the case of a 34-year-old patient with no significant medical history who was followed for primary membranous glomerulonephritis confirmed by renal biopsy, initially revealed by nephrotic syndrome and preserved renal function. After nine months of conservative treatment, the patient presented with progressive deterioration of renal function and increased proteinuria, leading to the initiation of immunosuppressive therapy. Persistent renal deterioration prompted a second biopsy, which revealed extracapillary proliferation in addition to membranous nephropathy. The clinical course was rapidly unfavorable, with oligoanuria, fluid overload, and respiratory distress requiring urgent hemodialysis. Despite optimal care and several dialysis sessions, no renal recovery was observed, and the patient progressed to end-stage renal failure. This case illustrates the rare occurrence and severity of crescentic transformation in primary membranous glomerulonephritis, highlighting the importance of close monitoring and rapid reassessment in the event of rapid disease progression.

## Introduction

Membranous glomerulonephritis, previously known as membranous nephropathy (MN) [1], is a glomerulopathy characterized by the formation of subepithelial immune deposits along the glomerular basement membrane, leading to diffuse thickening of the capillaries and structural alterations in the podocytes [2]. It is one of the main causes of nephrotic syndrome in adults, with variable clinical expression ranging from spontaneous remission to progression to chronic renal failure [3]. The global incidence of the disease is estimated at 8 to 10 cases per million individuals [4]. MN is histologically distinguished by the usual absence of endocapillary or extracapillary cell proliferation, which differentiates it from proliferative glomerulonephritis [5]. Glomerular crescents, consisting of extracapillary proliferation of parietal epithelial cells and macrophages in Bowman's space, are commonly seen in rapidly progressive glomerulonephritis and reflect severe glomerular damage [6]. Although their presence is exceptional in primary MN, isolated cases have documented the appearance of crescents in the absence of anti-neutrophil cytoplasmic antibodies (ANCA) or anti-glomerular basement membrane antibodies, suggesting a rare histopathological variant whose pathogenic mechanisms remain poorly understood [7]. We report a case of primary MN with positive anti-PLA2R antibodies in which crescent formation occurred despite the initiation of immunosuppressive therapy.

## Case presentation

A 34-year-old man from southern Morocco, with no significant medical history, presented in March 2024 to the nephrology department of Mohammed VI University Hospital in Marrakech, with a progressive generalized edematous syndrome, characterized by symmetrical bilateral edema of the lower limbs, facial puffiness, and ascites secondary to nephrotic syndrome with normal renal function.

At the initial clinical examination, blood pressure was 120/70 mmHg, and body mass index was 27 kg/m². No comorbidities were found apart from dyslipidemia. Laboratory tests showed hemoglobin of 13 g/dL, total cholesterol 2.3 g/L, LDL 1.6 g/L, HDL 0.43 g/L, triglycerides 1.04 g/L, serum creatinine 9 mg/L with an estimated glomerular filtration rate (GFR) 126 mL/min, and hypoalbuminemia 19.5 g/L. The 24-hour urinary protein excretion was estimated at 4 g/day. The urinary sediment was positive for microscopic hematuria.

The renal biopsy performed on March 22, 2024 revealed stage 1 MN. The anti-PLA2R antibody titer was positive at 500 U/mL, confirming the primary nature of the lesion. Conservative management was initiated, based on nephroprotective treatment with an angiotensin-converting enzyme inhibitor combined with lipid-lowering therapy and acetylsalicylic acid at a dose of 75 mg/day.

The clinical evolution was marked by a reduction in proteinuria to 1.2 g/day, but after nine months of nephroprotective treatment, renal function worsened, with serum creatinine rising to 63 mg/L, corresponding to an estimated glomerular filtration rate of 10 mL/min. This deterioration was accompanied by worsening of the nephrotic syndrome, characterized by the reappearance of generalized edema and a major increase in 24-hour proteinuria, from 1.2 g/day to 13 g/day.

The patient was readmitted to the hospital for a complete etiological assessment. Immunological testing revealed, for the first time, consumption of complement C3, which was confirmed in several subsequent tests with negative results for antinuclear antibodies (ANA), anti-native deoxyribonucleic acid (anti-native DNA), perinuclear and cytoplasmic antineutrophil cytoplasmic antibodies (p-ANCA, c-ANCA), and anti-glomerular basement membrane antibodies (anti-GBM). Control anti-PLA2R antibodies were positive at 300 U/mL (Table 1). Serology tests for hepatitis B, hepatitis C, syphilis, and human immunodeficiency virus were negative. Morphological investigations, including abdominal, cervical, and thoracic ultrasound, as well as abdominal-pelvic ultrasound, were normal.

**Table 1:** Laboratory results during the three hospitalizations Summary of biological and immunological parameters on admission for the three hospitalizations, highlighting the deterioration in renal function between each hospitalization despite therapeutic management. ANCA: antineutrophil cytoplasmic antibody; Anti-MBG: anti-glomerular basement membrane antibody

Parameters	At admission	2nd hospitalization	3rd hospitalization	Reference range
Serum creatinine (mg/L)	9	63	117	7-12
Urea (g/L)	0.28	0.95	1.95	0.19-0.54
Creatinine clearance (mL/min)	126	10	6	>90
Serum albumin (g/L)	19.5	12	24	35-50
24-hour proteinuria (g/24 h)	4	13	11.6	<0.15
Sodium (mmol/L)	137	133	143	135-145
Potassium (mmol/L)	4	3,59	3.7	3.5-4.5
Corrected calcium (mg/L)	90	92	76	85-105
Phosphorus (mg/L)	40	67	67	25-45
Bicarbonate (mmol/l)	20	17	10	22-28
ANCA	-	Négative	Négative	Négative
Anti MBG	-	Négative	Négative	Négative
Complement C3 (g/L)	1.06	0.88	0.6	0.9-1.8
Complement C4 (g/L)	0.38	0.4	0.3	0.1-0.4
Anti-PLA2R antibodies (U/mL)	500	300	-	Négative<14

Given the persistent and progressive nature of the nephrotic syndrome, the patient was classified as high risk for progression, prompting the introduction of a Ponticelli regimen. However, no clinical or biological improvement was observed. Serum creatinine continued to rise, reaching 117 mg/L, with persistent high proteinuria at 11 g/24 hours in December 2024. Given this rapid deterioration in renal function, a second renal biopsy was indicated. This was performed in the same month and revealed extracapillary proliferation in addition to primary MN.

**Figure 1:**
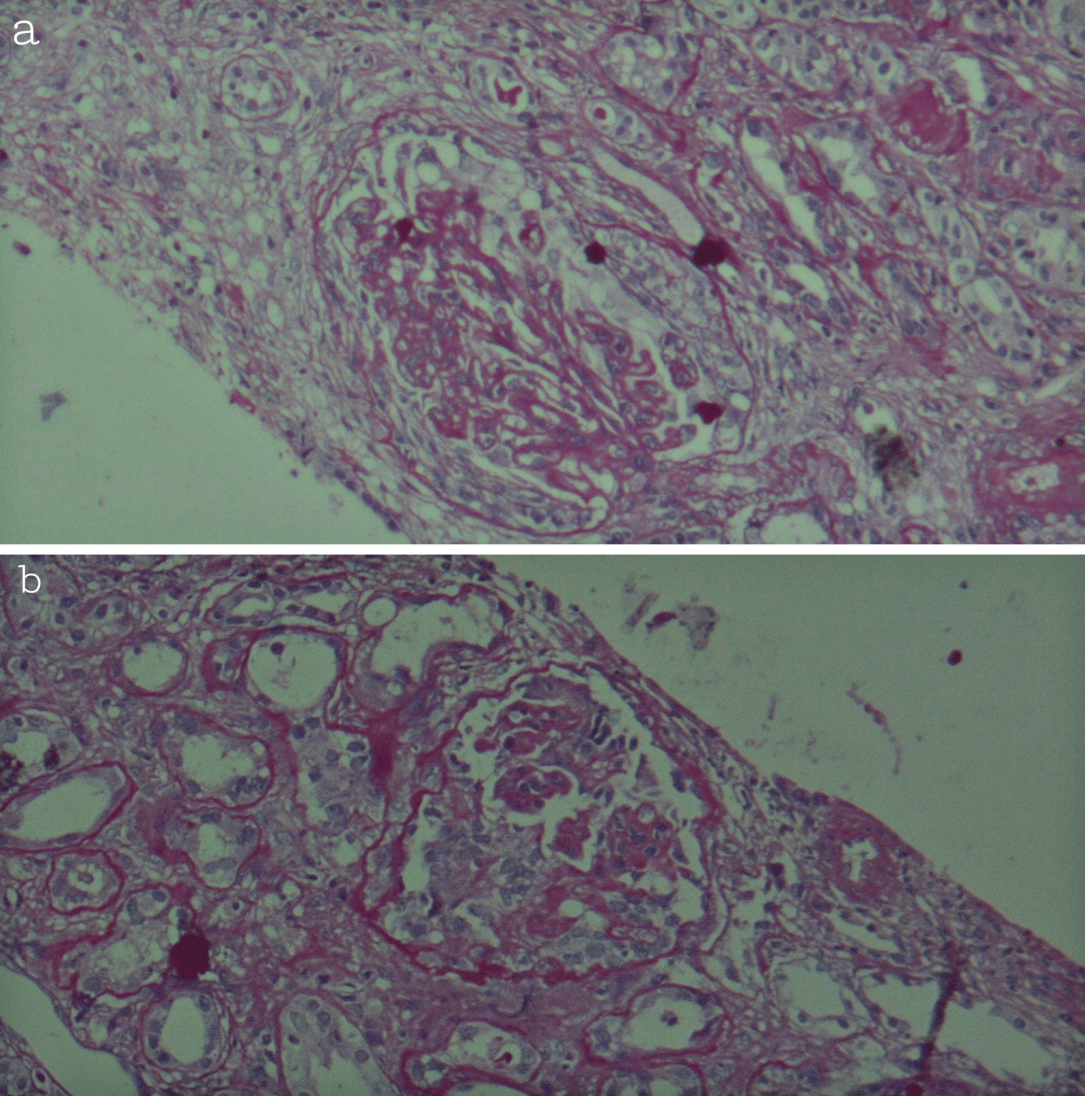
(a, b) Histological sections stained with periodic acid-Schiff (PAS) reaction, viewed at ×40 magnification, showing moderate interstitial fibrosis and moderate tubular atrophy

**Figure 2:**
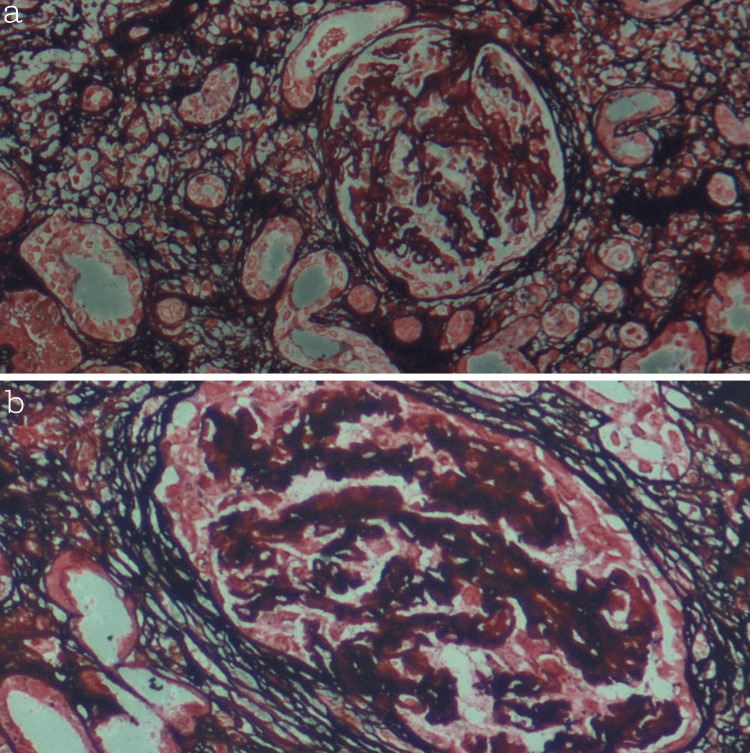
(a, b) Sections stained with JMS (Jones Methenamine Silver), viewed at ×10 magnification, showing extracapillary proliferation (crescent formation) with distortion of glomerular architecture and basement membrane irregularities

**Figure 3:**
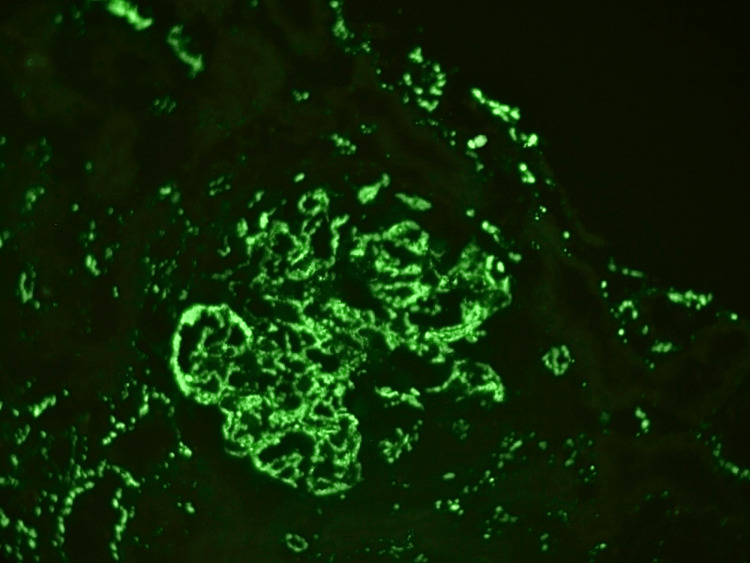
Direct immunofluorescence analysis showing granular deposits of immunoglobulin G

The histological findings showed membranous glomerulonephritis with partial circumferential cellular crescents (33%), moderate interstitial fibrosis around 30%, and moderate tubular atrophy (35%) (Figures 1-3).

In this context of rapid deterioration, the course of the disease was marked by the onset of anuria, significant fluid overload complicated by acute pulmonary edema and respiratory distress, requiring the urgent initiation of hemodialysis. Treatment with pulse of Solumedrol combined with intravenous cyclophosphamide was initiated.

Despite optimal care and several hemodialysis sessions, no recovery of urine output or renal function was observed. The combination of prolonged dependence on dialysis, lack of functional recovery, and severe histological lesions observed at rebiopsy led to the consideration of progression to end-stage renal failure. The patient therefore underwent arteriovenous fistula creation and the placement of a permanent vascular access for continued chronic hemodialysis.

## Discussion

Primary MN is an autoimmune glomerulopathy characterized by the presence of anti-PLA2R antibodies, responsible for granular subepithelial deposits and often massive proteinuria, which can progress to chronic renal failure [8]. The appearance of extracapillary proliferation in primary MN is a rare phenomenon, described mainly in isolated observations or small series of patients with primary MN associated with anti-PLA2R antibody positivity and in the absence of ANCA or anti-MBG antibodies [7]. The proportion of glomeruli affected by crescents varies between 20 and 30% in these series. Their presence is generally associated with rapid deterioration of renal function and persistent nephrotic syndrome [9].

Our patient presented with primary MN, confirmed by anti-PLA2R positivity at 500 U/mL, initially with nephrotic syndrome and stage I on biopsy. In our patient, re-biopsy revealed 33% of glomeruli with partial circumferential cellular crescents, accompanied by moderate interstitial fibrosis (30%) and tubular atrophy (35%). These histological features correspond to observations reported in the literature, where crescents are associated with an unfavorable renal prognosis, often requiring the rapid initiation of aggressive immunosuppression [7]. Initial treatment with the Ponticelli regimen in our case did not control the nephrotic syndrome or prevent the progression of renal failure, illustrating the possible refractoriness of MN with transformation into an extracapillary proliferative form, particularly in cases of high immunological load and advanced histological involvement, a situation also highlighted by the study by Wang et al. [10]. Our experience with this patient highlights the need for rapid reassessment when the course of nephrotic syndrome is atypical, as there may be a more insidious explanation for the patient's sudden decline despite treatment.

The pathophysiological mechanisms underlying this extracapillary proliferative transformation in primary MN remain poorly understood. It has been suggested that persistent complement activation, particularly via the classical and alternative pathways, as evidenced by decreased C3 levels, leads to extracapillary proliferation of parietal epithelial cells, resulting in crescent formation and rapid loss of functional nephrons [6]. In our observation, the repeated consumption of C3 in the absence of other autoantibodies reinforces the hypothesis of such a pathophysiological mechanism.

The prognosis for primary MN with crescents remains generally poor, especially if a large proportion of glomeruli are affected, and renal function is already impaired at the time of diagnosis. Li et al. reported that a significant proportion of these patients progress to end-stage renal failure despite conventional immunosuppressive treatment [9]. Our case illustrates this progression, requiring the initiation of chronic hemodialysis.

## Conclusions

The appearance of extracapillary proliferation in primary MN is a rare but severe histopathological complication, correlated with rapid deterioration of renal function and persistence of nephrotic syndrome. The failure of the Ponticelli protocol in this case highlights the possible refractoriness of the disease when the immunological burden is high and histological involvement is advanced. This observation highlights the need for close monitoring of patients with rapid progression, early re-biopsy to confirm lesion transformation, and prompt consideration of alternative therapeutic strategies, including targeted immunotherapies. Finally, these data illustrate the importance of further studies aimed at elucidating the pathophysiological mechanisms of extracapillary proliferation in primary MN in order to optimize the management and prognosis of high-risk patients.
